# Glucose homeostasis dependency on acini–islet–acinar (AIA) axis communication: a new possible pathophysiological hypothesis regarding diabetes mellitus

**DOI:** 10.1038/s41387-018-0062-9

**Published:** 2018-10-08

**Authors:** Stefan G. Pierzynowski, Peter C. Gregory, Rafał Filip, Jarosław Woliński, Kateryna Goncharova Pierzynowska

**Affiliations:** 1 Department of Biology, Lund University, Sölvegatan 35, 22362 Lund, Sweden; 2 Anara AB/SGPlus, Alfågelgränden 24, 23132 Trelleborg, Sweden; 3 PROF/Vitanano Sp.z o.o., Woronieckiego 1a-13, 20491 Lublin, Poland; 4 Department of Medical Biology., Inst, Rural Medicine, Jaczewskiego 2, 20950 Lublin, Poland; 5 Innovation Centre - STB, Skarszewska 23, 83100 Tczew, Poland; 6 Department of Gastroenterology with IBD Unit of Clinical Hospital 2, University of Rzeszow, Lwowska 60, Rzeszow, 35301 Poland; 7 Department of Animal Physiology, The Kielanowski Institute of Animal Nutrition and Physiology Polish Academy of Sciences, Instytucka 3, 05110 Jabłonna, Poland

## Abstract

Studies have highlighted the existence of two intra-pancreatic axes of communication: one involved in the regulation of enzyme production by insulin—the insular–acinar axis; and another involved in the regulation of insulin release by pancreatic enzymes—the acini–insular axis. Previous studies by our laboratory show that pancreatic enzymes can affect blood glucose homeostasis and insulin secretion independently of their digestive functions, both from the gut lumen and probably from the blood. As a result we would like to introduce here the concept of acini–islet–acinar (AIA) axis communication (feedback), which could play an important role in the development of obesity and diabetes type 2. The AIA feedback links the endocrine and exocrine parts of the pancreas and emphasizes the essential role that the pancreas plays, as a single organ, in the regulation of glucose homeostasis by amylase most probably in gut epithelium and by insulin and glucagon in peripheral blood.

## Introduction

The co-existence of the endocrine and exocrine parts of the pancreas has prompted years of investigation into the functional relationship between the two parts. From both a physiological and clinical point of view, it is easier to study these two parts of pancreatic function separately. Thus, the exocrine pancreas has always been viewed as part of the gastroenterology domain, whereas the endocrine pancreas has been an area of interest for those studying diabetes, as described in the aims and scopes of leading scientific journals in the respective fields^1,2^.

Nevertheless, although exocrine tissue forms the majority of the pancreatic mass, whereas the endocrine cells are “contained” in well-defined, encapsulated islets, the endocrine part of the pancreas is actually phylogenetically older than the exocrine part^3^. Moreover, it was also shown in mouse embryogenesis that the first pancreatic cells formed from the dorsal buds are glucagon-producing cells^4^. The effect of glucagon on exocrine secretion has not yet been fully elucidated. Some studies have demonstrated inhibitory effects of glucagon on secretin- and cholecystokinin-stimulated pancreatic protein but not on bicarbonate secretion^5,6^, others showed that glucagon inhibits both postprandial protein and bicarbonate secretion^5,7^. However, more recent studies indicate that the effect of glucagon on isolated pancreatic acini appears to be direct and stimulatory, instead of inhibitory, suggesting complex action at the cellular versus physiological levels, with the inhibitory effects perhaps involving somatostatin release^5,8^. Thus, one might conclude that the α- and/or β cells are phylogenetic precursors of the mammalian pancreas and, that the exocrine pancreas might have formed through the actions of the pancreas-specific transcription factor 1 (PTF 1). PTF 1 is a signaling factor that regulates the expression of exocrine-specific genes, in endocrine pancreatic cells. Previous studies in animal models have shown that downregulation of PTF 1 in pancreatic exocrine cells may lead to their transformation into endocrine cells^3,9^.

The above-mentioned example shows that inter-pancreatic cell signaling via extracellular fluid is possible, and what is also of importance here is that the end products of both the endo- and exocrine parts of the organ may interact locally. Second, the above-mentioned concept was further developed in human studies, which led to the general conclusion that exocrine cells from the extracted human pancreas can be reprogrammed into transplantable insulin-producing cells that may be fully metabolically active. The underlying mechanism is based on the fact that triggering mitogen-activated protein kinase (MAPK) and signal transducer and activator of transcription 3 (STAT3) signal transduction can transform them to β-like cells^10^. Moreover, ectopic signaling through MAPK and STAT3 might convert human acinar cells to β-like cells as well. Other studies have confirmed this hypothesis and have also shown that ectopic expression of activated MAPK and STAT3 in human pancreatic acinar cells may activate the proendocrine transcription factor neurogenin 3, resulting in the reprogramming of human acinar cells to insulin-positive β-like cells, which are able to ameliorate chemical diabetes^11^. There is therefore clear evidence that complex inter-relationships between pancreatic exocrine and endocrine tissue exist or can be activated.

In fact, there is compelling evidence for an islet–acinar axis, which suggests that insulin and other factors derived from the pancreatic islets directly regulate and may even be essential for normal pancreatic acinar cell function^12–19^, especially that of amylase production, a situation that is supported by the “halo phenomenon”^20–22^. On the other hand, it is likely that hyperstimulation of the pancreatic acini cells during long-term hyperinsulinemia (obesity—diabetes type 2 (DT2)), and possibly other islet hormones as well, which come into contact with the acini^23^, can attenuate pancreatic enzyme production. Thus, in the obese Zucker rat it has been shown that long-lasting hyperinsulinemia and insulin resistance results in impaired amylase gene expression in the exocrine pancreas and destruction of amylase production^24^. Moreover, there is evidence that there may be a similar response in humans, as in non diabetic, obese women an increase in body mass index (BMI) has been shown to correlate with loss of pancreatic exocrine function^25^, whereas a high percentage of patients with DT2 (28–54%) have been reported to develop exocrine pancreatic insufficiency^26^.

Looking at the reciprocal relationship, a strong association has been shown to exist between exocrine pancreatic insufficiency (EPI) and loss of β-cell function: thus diabetes mellitus was observed in 70% of patients with chronic pancreatitis (CP) and in 91% patients with chronic calcifying pancreatitis, whereas only 16% CP patients also had hypoglucagonemia^27^. The latter fact could highlight possible changes in the insulin–glucagon relationship taking place during CP. In healthy status as well as in juvenile diabetes type 1 (DT1), insulin and glucagon have mutual reciprocal regulation (for review see Ungern and Cherrington^28^). In conditions of DT1, α cells produce high levels of glucagon, whereas insulin production is deficient owing to β-cell loss^29^. In states of obesity/DT2, when insulin production is upregulated, glucagon secretion should be downregulated, however, the opposite situation is observed, another aspect of the “enigma” concerning the mechanisms controlling glucagon secretion^4^. Perhaps, consideration of the AIA concept might help to to solve this puzzle. Amylase supplementation (both intraduodenally and intravenously) decreases insulin levels similarly to glucagon action^30^. This raises the possibility that amylase might decrease both insulin and glucagon levels and, as a consequence, blood glucose level. It will be necessary to design specific studies to try to clarify insulin–amylase–glucagon relations in health and obesity.

The onset of diabetes may at least partly be due to loss of β cells in CP, perhaps because of the loss of trophic factors from the acini, as well as loss of responsiveness to glucose^31^ and hypersecretion of glucagon^32,33^. Patients with EPI secondary to cystic fibrosis also show decreased β cell secretory capacity compared with pancreatic-sufficient cystic fibrosis patients, in this case there is evidence of impaired proinsulin processing^34^. Diabetes secondary to CP is also associated with an increased meal-induced secretion of somatostatin compared with patients with type I diabetes or controls^35^, whereas oral glucose induces a rise in glucagon instead of the fall normally seen in controls, as well as a larger rise than normal in GIP^36^.

It has been known for many years that pancreatic enzymes can be detected in systemic blood in both healthy as well as diseased individuals, and moreover the plasma enzyme concentrations can be used as a measure of pancreatic function^37^. Nota bene! absorption of pancreatic enzymes from the intestine, under physiological conditions, is negligible and in healthy individuals should not take place at all^38^. Furthermore, the presence of immunoreactive trypsinogen, as well as amylolytic and lipolytic activity in the blood, suggests that plasma levels are a result of “leakage” of pancreatic enzymes from the pancreas into the bloodstream, rather than re-absorption of the secreted enzymes^37,39^. It is logical that in such circumstances, the concentration of pancreatic enzymes in the interstitial fluid surrounding the pancreatic acini and nearby islets would be many times higher than that in the peripheral blood. Direct investigation into the impact of the exocrine pancreas on endocrine pancreas function is complicated and requires the use of specific experimental models. Recent studies by our laboratory, performed on the EPI pig model, showed a strongly attenuated insulin response following both intravenous and oral glucose challenge tests^40^, in agreement with the earlier mentioned clinical observations. We found that the EPI pigs have involuted pancreatic acini with a reduced level of pancreatic enzyme production/secretion, and that the “surrounding” endocrine pancreatic islets produce less insulin compared with that observed in healthy, control pigs^40^. These results led us to suggest the possible existence of an intra-pancreatic, acini–islet axis communication^40^, which may be necessary for the full function and possibly also the health of the β cells.

The pancreatic enzymes (or other contents of the exocrine secretions) may “directly”—in first pass—exert trophic or other mediatory effects on the pancreatic islet cells, resulting in a reduced or improved insulin response. One possible mediator could be MAPK, as this has been proven to play a critical role in the adaptive responses to thermal, osmotic, and oxygen stresses, including β cells responses to the glucose overload^41^. It is well established that increased MAPK activity is not only required for the initiation and maintenance of pancreatitis, but it has also been hypothesized that the activation of MAPK might be triggered by pancreatic enzymes (amylase)^42^. Perhaps low enzyme concentrations may exert positive and stimulatory effects on β cells via slight MAPK activation, whereas high concentrations may trigger the inflammatory cascade and lead to the depletion of pancreatic function. The influence of acini–islet axis communication may be extended further if we consider also that extra-digestive properties of the pancreatic enzymes may influence glucose homeostasis while acting from the gut or systemic circulation. It is well known that the growth and maturation of the intestinal mucosa is moderated/accelerated by exogenous pancreatic or bacterial proteinases^43^, whereas elevated proteinase activity in the adult gut can increase para-cellular gut permeability, thus deteriorating intestinal barrier function^44–46^. Recent findings suggest that glucose/insulin homeostasis can be partially regulated by extra-digestive properties of enterally applied pancreatic amylase and protease^47,48^. Further studies show that intravenous (iv) infusion of amylase reduces the production of insulin and C-peptide, without influencing glucose tolerance in response to an iv glucose load^30^, showing there may be an extended acini–islet feedback mechanism.

Hence, we would like to introduce here the hypothesis that the islet–acinar and acini–islet axes both serve as feedback loops. The system is balanced (fasted state?) by a quasi threshold level concentration of pancreatic hormones and enzymes (possibly amylase and its peptides) in the interstitial fluid surrounding the islets and acini, but is influenced as well by enzymes in the blood and in the gut lumen and high levels of amylase can reduce insulin release.

## Mutual interplay between gut hormones and amylase

Although the trophic effect of insulin on the peri-insular acini is well described, the role of incretin hormones in the interplay between pancreatic acinar and β cells has not yet been fully elucidated. However, it is commonly accepted that GLP-1 does not directly stimulate amylase release. On the other hand, novel data indicate that GLP-1 can trigger phosphorylation of the epidermal growth factor receptor and activation of Foxo1 (Forkhead box protein 01), which may result in cell growth with concomitant enzyme release^49^. Based on the aforementioned observation, one might conclude that GLP-1-induced signaling pathways play an important role in the stimulation of the exocrine pancreas and thus in increasing amylase levels^49^. In addition, it has been shown in patients with EPI owing to CP, that pancreatic enzyme substitution increases serum of another incretin hormone—glucose-dependent insulinotropic polypeptide (GIP). This effect could be attributed either to the direct influence of amylase on GIP-producing cells, and/or to the enhanced digestion and absorption of nutrients stimulating the secretion of GIP^50^. These observations were confirmed by Knop et al. in 2006^51^, and extended to show that postprandial GLP-1 level was also increased by enzyme supplementation, suggesting that the same mechanisms might also be in play with GLP-1. Other pancreatic hormones such as pancreatic polypeptide and somatostatin exert inhibitory effects on pancreatic exocrine secretion, at least partly via inhibition of insulin release, whereas the influence of glucagon remains unclear at present^19^. A study performed in asymptomatic middle-aged adults showed low serum amylase levels were accompanied by high leptin levels as well as an increased HOMA-IR^52^. This could, at least in part, be attributed to the fact that increased insulin secretion may lower insulin responsiveness by downregulating insulin receptor expression or inhibiting insulin signaling within pancreatic acinar cells^51^. On the other hand, it must be pointed out that the role of amylase in the control of gut hormones has not yet been elucidated.

## Pancreatic enzymes in glycaemic control

The fact that insulin produced in the pancreatic islets affects enzyme production in the pancreatic acini has been known for many years^15,18,53,54^. There is also a considerable amount of evidence—albeit not fully recognized—which suggests the involvement of pancreatic enzymes in glycaemic control. For example, we have proven that enteral administration of acinar enzymes help to control insulin production and/or release in response to a glucose challenge^40^. Recently, we have shown that pancreatic enzymes have extra-digestive properties at the gut level, which are involved in the regulation of glucose absorption and metabolism; oral administration of a pancreatic enzyme supplement 1h prior to an oral or meal glucose tolerance test (GTT) lowered blood glucose, whereas after an iv GTT glucose elimination was slowed together with reduced insulin response to the challenge^48^. The response may be owing to amylase as intestinal infusion of pancreatic amylase or amylase-derived peptides similarly lowered blood glucose to intestinal glucose challenge^48^. Interestingly, enterally administered microbial amylase was also found to lower the insulin response required for glucose utilization during an iv glucose tolerance test^47,48^. Taking into account other studies where reduced blood amylase concentrations were associated with glucose intolerance, we speculate that the mechanism behind these findings is based on the specific signal transduction of enteral or parenteral amylase or amylase-derived components/peptides, which interact with the glycoconjugates—possibly receptors on the apical or basolateral surface of the enterocyte^48^. Pancreatic amylase has been reported to bind to *N*-glycans in intestinal brush border membrane and inhibit intestinal glucose absorption by SGLT1^55,56^. Furthermore, amylase and/or amylase derivatives could enhance glucose uptake in insulin-independent tissues such as enterocytes via GLUT1 or GLUT2, and in this case could serve as a defensive mechanism against the development of hyperglycemia. We suggest that amylase limits insulin secretion by directing glucose from the bloodstream to the intestine; overall gut amylase lowers glucose absorption and insulin release. Thus, glucose utilization by the enterocytes during its first passage to the bloodstream or the depletion of glucose from the bloodstream can be recognised as one of the insulin-independent aspects of glucose metabolism controlled by amylase. This aspect is probably not functioning as it should in the obese state.

By making use of a duodenal–jejunal bypass pig model, we found that the spatial separation of pancreatic enzymes, which takes place owinig to the intestinal rearrangement causes an increase in blood amylase activity^30^ and facilitates the clearance of glucose from the blood following glucose challenges^48^. Data in pigs also support previous findings in the rat Roux-en-Y Gastric Bypass model^57^, in which removal of the duodenal section of the intestine significantly alters the morphology of the remaining intestine and reduces glucose transport function. Taking into account the impact of amylase administration on glucose disposal, we hypothesized that intestinal rearrangement in bariatric surgeries might be at least partially related to the increase of amylase activity in both the biliopancreatic limb and in the blood. In our studies, the duodenal–jejunal bypass pigs exhibit increased blood amylase concentrations compared with that observed in sham-operated pigs^30^. This observation suggests a new, qualitative role of amylase in glycaemic control.

At the same time, several studies have reported that an increased proteolytic activity attenuates the binding of insulin to its receptor^58,59^. Stimulation and overproduction of proteinases can contribute to increased insulin resistance, indeed enteral administration of proteinase has been found to increase insulin release to a glucose challenge with subsequent reduction of insulin sensitivity^47^. However, the activity of intestinal proteinases, in particular trypsin, could be essential for the cleavage of amylase and the subsequent release of its functionally active components at the level of the gut.

The world obesity epidemic is coupled with the overconsumption of carbohydrates^60,61^. Thus, it became more important, albeit more complicated owing to methodological issues, to explore the consequences of the non-digestive actions of pancreatic enzymes on insulin and glucose metabolism, via the gut mucosa. We decided that the best model to use for investigating non-digestive effects of pancreatic enzymes on insulin and glucose metabolism was the classical bariatric surgery pig model^48^. Bariatric surgery has been shown to improve postprandial glucose concentrations and eliminate DT2 in obese patients^62^, thus, suggesting an indirect, gut-driven mechanism. From a physiological point of view some of the most effective types of bariatric surgery limit the availability of pancreatic enzymes for food digestion^63^. Pancreatic enzymes in the so-called biliopancreatic limb, which is formed during classical bariatric surgery, could be auto-digested. In some publications, successful bariatric surgery has been compared with a status of exocrine pancreas insufficiency, characterized by the malabsorption of nutrients^64^. Thus, we believe that the bariatric pig model serves as an appropriate model for studying the relationship between gut pancreatic enzymes and glucose absorption and insulin release.

In addition, elimination of pancreatic enzymes from the biliopancreatic limb (auto digestion) essentially lowers the absorption of glucose following oral glucose loads. We speculate that the decreased glucose absorption could also be linked to the presence of active salivary amylase in the alimentary and common limbs. Salivary amylase passing through the stomach in substrate bound form comes into contact with a very low level of pancreatic enzyme proteolytic activity in the intestine of bariatric pigs and thus it is not destroyed. This hypothesis is supported by recent experiments by Date et al.^55,56^ as well as by some earlier findings^65^.

In addition, observations from our laboratory^16,17,66,67^ indicate there is increased secretion of alpha- amylase from the exocrine pancreas in milk-fed pigs. Thus, we posed the question, “what role does amylase play, because there is no substrate (starch) for that enzyme in mothers’ milk?” We could not answer the question at that time! Now, we can hypothesize that neonatal pancreatic amylase has a role in the regulation of blood glucose. Insulin production in neonate piglets is minimal—below detection level—however, blood amylase concentrations are high—600 U/L^30^. Despite minimal insulin production, postprandial blood glucose concentrations, as well as nutrient assimilation in the neonate are perfectly regulated^23,68^. The presence of high blood levels of amylase raise the possibility that if our hypothesis is correct, amylase might also influence glucose metabolism (GLUT1/GLUT2 in other tissues, such as liver, kidney).

The hypothesis presented in this paper is supported by the studies of Mandel and Breslin (2012), which showed that high endogenous amylase activity within the blood is associated with improved glycaemic homeostasis^69^. Conversely, low serum amylase concentration is associated with an increased risk/prevalence of metabolic syndrome^70,71^ raised BMI, as well as insulin resistance and decreased plasma insulin concentrations even after adjusting for BMI^52^. An intriguing study done by Nakajima and Magee in 1970^72^, showing that pancreatic exocrine secretion, especially amylase secretion was significantly inhibited following a rapid iv infusion of a 40% glucose solution^72^, is another indication of a role for amylase in glucose homeostasis.

Furthermore, a clear negative correlation has also been observed between salivary amylase secretion and obesity^73,74^. Thus, in Finland, obesity in girls was found to coincide with low copies of the salivary amylase gene^73^. Conversely, a high copy number of the salivary amylase gene was shown to have a beneficial role as an anti-obesity factor in Mexican children^74^. Individuals with a high copy number of the amylase gene produce more salivary amylase in comparison with individuals with a low copy number of the amylase gene and are thus able to absorb more glucose, which in turn can be converted to fat. But this would be expected to lead to obesity—not protect against obesity, therefore there must be another explanation.

The evidence presented above led us to suggest the possibility of a sustained role of the AIA axis in the regulation of blood glucose homeostasis. High concentrations of alpha-amylase within the gut (blood) could be a factor regulating glucose absorption and utilization from/in the gut and subsequent glucose disposal, in an insulin-independent manner within the body that does not lead to fat deposition, causing indirectly and perhaps also directly a reduction in insulin secretion.

Thus, pancreatic enzyme production and the “health” of the pancreatic acini are essential for insulin release and enzymes involved in regulation of insulin production are necessary for efficient glucose metabolism. When the islet–acinar and acini–islet axes are coupled together the overlooked feedback mechanism, which regulates both insulin and pancreatic enzymes is evident. However, the biological consequences related to the regulation by the AIA axis have not previously been outlined.

## Deterioration of AIA axis feedback disrupts glycaemic control

**Fig. 1:**
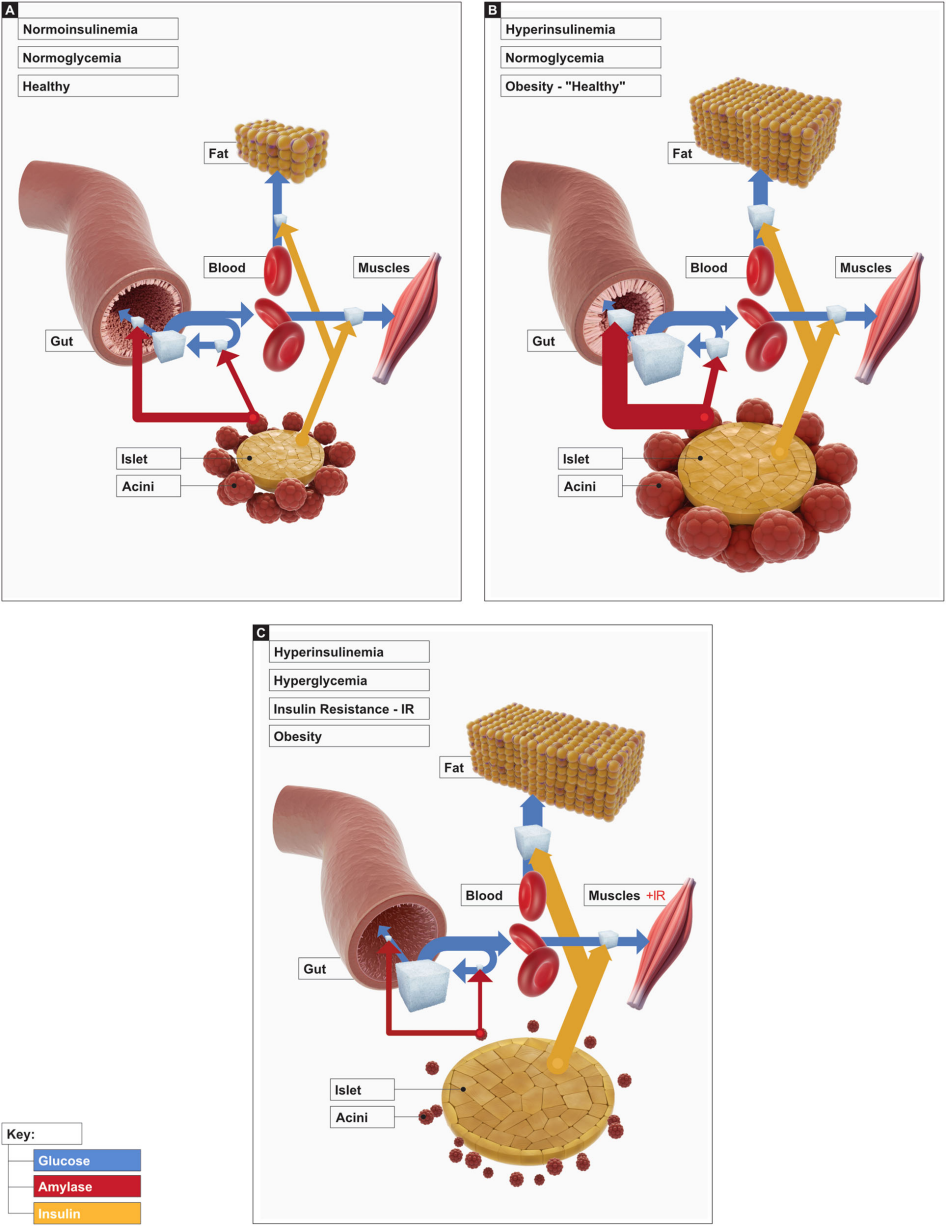
Role of AIA in the development of obesity and diabetes **a** Balanced Acini–islet–acinar (AIA) feedback maintains sustainable glucose utilization by insulin-independent (muscle, fat, etc.) and amylase-dependent glucose assimilation (gut tissues, gut bacteria). **b** The overstimulation of the pancreas enlarges the AIA defensive mechanism against the hyperglycaemic condition; **c** exhaustion of the acinar cells leads to the development of functional exocrine pancreatic insufficiency and hyperglycemia as a consequence

An understanding of the metabolic impact of the pancreas, acting as a single organ can be essential in the context of obesity development. The impact of the exocrine pancreas on the development of insulin resistance can be schematically described as a feedback loop (Fig. 1a–c). In conditions of good health and moderate consumption of digestible carbohydrates, both parts of the pancreas operate at an optimal level. The AIA regulation is balanced and can properly regulate glucose absorption, redisposition, and metabolism in particular tissues. A relatively small part of the dietary glucose consumed is used up by the intestinal cells including that for enterocytes’ or for gut bacterial glycogen production^75,76^ glycogen production (Fig. 1a).

When dietary glucose/carbohydrate consumption increases, both the exocrine and endocrine pancreas increase their activity to ensure digestion and glucose disposal. Therefore, in the above-mentioned, transient stage, intestinal glucose consumption might also increase (Fig. 1b). However, the presence of several factors will define actual health status. The consumption of sugars will stimulate an increase in amylase and insulin production and in so doing postprandial hyperglycemia will be downregulated. However, when individuals develop obesity owing to overconsumption of simple sugars, which per se stimulate insulin secretion (via incretins or directly), the hyperinsulinemia causes hypersecretion of pancreatic enzymes. This has been proven in a study using cows, in which the intraduodenal loading of glucose, after fasting, resulted in significantly increased amylase secretion^77^. Some patients (who very seldom visit their physicians) could still display a “healthy reference range” when assessing liver, cardiovascular, etc., system/organs function. These patients would only present with hyperinsulinemia and an increased BMI. In such cases, amylase, through its extra-digestive action, participates in the assimilation of glucose in insulin-independent tissues, e.g., gut mucosa. However, the presence of “free” insulin in the blood enhances the development of insulin resistance. At the same time, the presence of pancreatic proteinases in the blood could provoke the development of insulin resistance^58,59^.

Further overstimulation of pancreatic factors will lead to the development of metabolic syndrome and DT2 (Fig. 1c). These patients are most likely still consuming a large amount of carbohydrates. However, the possibly attenuated/fatigued AIA regulation exhausts pancreatic enzyme production, or in insulin resistance the acini no longer respond to insulin. Thus, the patients exhibit symptoms of functional exocrine pancreatic insufficiency—as the overproduction of insulin can exhaust acini cell function (enzyme production). A lot of the glucose consumed is thus absorbed and redistributed predominantly to fat synthesis owing to the increased tissue fasting and impaired insulin control. However, all the changes described above can cause pancreatic involution and interruption of proper acini function, which impacts the insulin response, leading to the development of exocrine and endocrine pancreatic insufficiency as a result of most probably impaired pancreatic enzyme secretion.

Notwithstanding the appearance of novel therapies for DT2, including incretin drugs, the natural course of the disease demands the introduction of insulin. This is the consequence of islet β-cell failure, which is preceded by peripheral insulin resistance. Thus, the hypothesis that enteral amylase may enhance the reduction of glucose absorption by directed glucose utilization in the gut mucosa and in that way lower insulin release, is attractive and worth consideration.

**Fig. 2:**
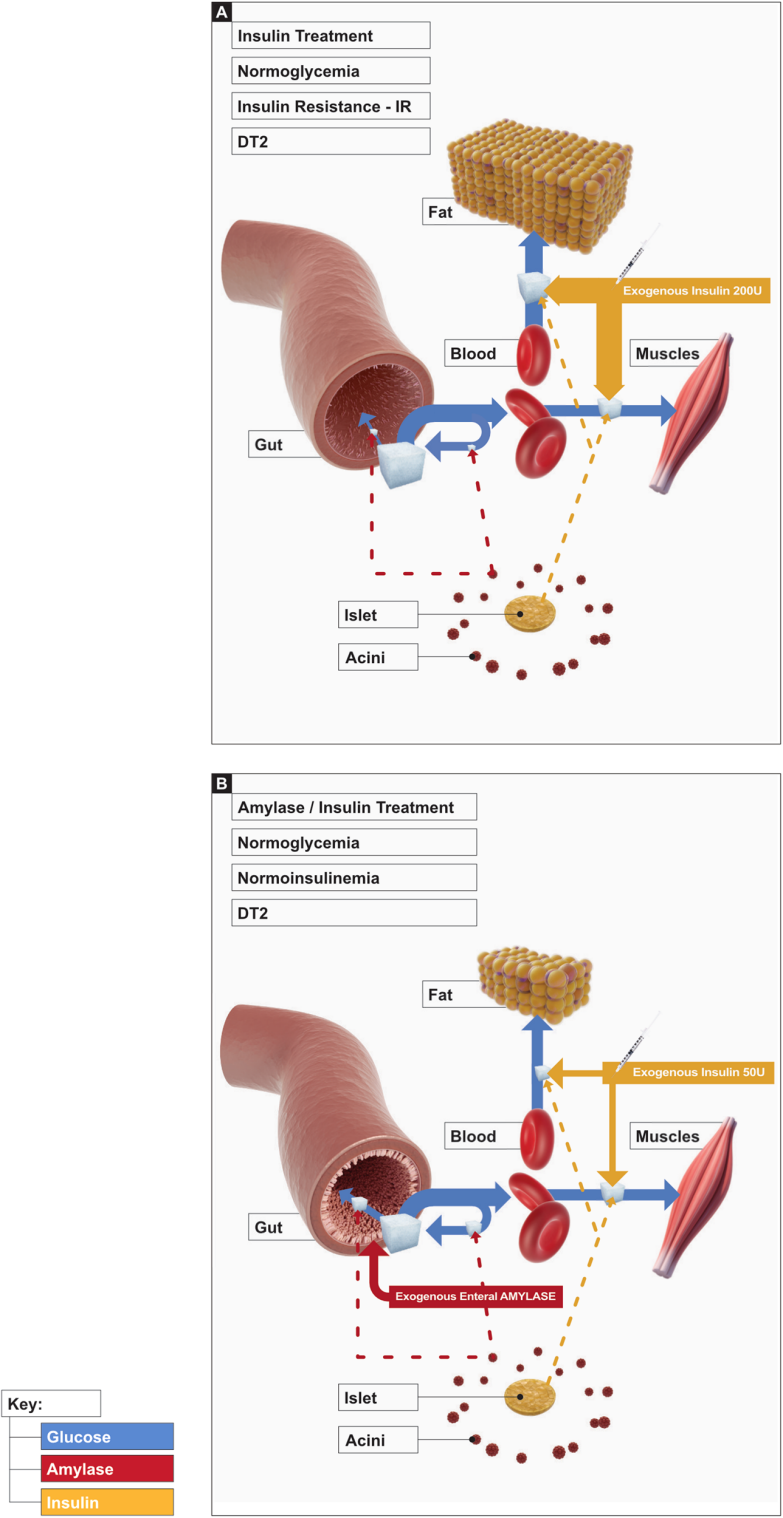
The classic DT2 treatment **a** D2 treatment against the hyperglycaemic condition involves insulin therapy, which does not fully restore pancreatic function and correct (causing obesity) distribution of glucose. Combined insulin–amylase therapy as a possible future treatment **b** mimics proper pancreatic regulation over glucose metabolism in different compartments

When T2D has fully developed, insulin therapy is prescribed (Fig. 2a). However, it seems possible that the administration of oral amylase at that stage might effectively support the insulin therapy, directing glucose metabolism to the intestine (Fig. 2b), which could essentially lower the amount of insulin required. The possibility that oral amylase treatment at the stage of hypoinsulinemia and hyperglycemia (Fig. 1c) could postpone or prevent the transition to complete DT2 should not be overlooked.

In summary, the non-digestive actions of pancreatic enzymes in the gut presented in the current paper extend the hypotheses, highlighting the central role of the pancreas, acting as a single organ, in obesity and DT2 development. The proposition of using amylase to delay/prevent obesity/DT2 development to treat these conditions in a clinical setting is novel, but seems to be quite rational.

Our hypothesis postulates that enteral amylase could be responsible for reducing the amount of glucose absorption into the blood and as a consequence, insulin release is lowered. Results in pig studies^48^ indicate that the gut, triggered by amylase or its peptides, actively participates in regulating the amount of glucose absorbed into the blood and that which is metabolized by the gut tissues (e.g., enterocyte metabolism/turnover), so limiting the availability of glucose for central metabolism. The presence of amylase or amylase-derived peptides in the gut reduces glucose absorption into the systemic blood and at the same time also limits insulin release, providing evidence for the existence of a gut-derived amylase-dependent mechanism, which regulates blood glucose concentrations in an insulin-independent manner.

A thorough understanding of the regulatory mechanisms involved in the above-mentioned phenomenon could be a milestone in the fight against obesity. Glucose will not stimulate insulin release and be converted to fat without being absorbed into the bloodstream. In addition, the glucose that is not absorbed will not provoke the development of T2D or insulin resistance!

We propose that the use of amylase, as a factor to slow down the development of or to treat obesity and diabetes, should be seriously considered. After all, the gut per se (mucosa) is perhaps the third main glucose-metabolizing compartment of the body, in addition to muscle and fat, albeit, we suggest, in an amylase-dependent, insulin-independent manner!
